# Meiotic Spindle View improves the Outcome of IVF in Poor Responders: A Retrospective Analytical Study from an Indonesian IVF Center

**DOI:** 10.5935/1518-0557.20240006

**Published:** 2024

**Authors:** Binarwan Halim, Hilma Putri Lubis, Ichwanul Adenin, Jesselyn Angellee, Rizky Senna Samoedra

**Affiliations:** 1 HFC IVF center Division of Reproductive, Endocrinology and Infertility Department of Obstetrics and Gynecology, Faculty of Medicine, Universitas Sumatera Utara, Medan, Indonesia

**Keywords:** poor responder, meiotic spindle view, IVF, oocyte

## Abstract

**Objective:**

Previous studies have shown that the meiotic spindle is not always aligned with the first polar body (PB) in metaphase II human oocytes. Polarized Light Microscopy (PLM) has been used to observe and locate the meiotic spindle to avoid disrupting it while injecting oocytes. The aim of this study is to evaluate the relationship between meiotic spindle view and IVF-ICSI outcomes in poor responder women.

**Methods:**

This study was a retrospective analytical study, carried out from January to November 2019; involving 115 poor responder women who underwent IVF-ICSI cycles at the Halim Fertility Center, Indonesia. The patients were divided into two groups: group I without meiotic spindle viewing (non-MSV) as control group, and group II with meiotic spindle viewing (MSV) as the case group. The meiotic spindles were imaged before ICSI with Oosight microscopy. Baseline characteristics and IVF-ICSI outcomes of both groups were compared.

**Results:**

Our study included 115 poor responder women with non-MSV group (71 women), and an MSV group (44 women). The results showed that there was no significant difference in the fertilization rate between the two groups (*p*>0.05), but the embryo cleavage rate was higher in the MSV group when compared to the non-MSV group; and there was a significant difference between the two groups (*p*<0.05). The fertilization rate was higher in aligned than misaligned spindle and there was a significant difference between the two groups (*p*<0.05). Good quality embryo was higher in MSV group than non-MSV group (59.05% *vs*. 63.95%).

**Conclusions:**

Meiotic spindle view might improve the outcome of IVF in poor responder women.

## INTRODUCTION

Poor response is one of the most challenging problems in IVF. It is estimated that 9-24% of IVF patients are poor responders who are often described as infertile women with the criteria of having few oocytes (< 3 follicles), advanced age (> 38 years old), decreased follicular response and low estradiol level (Masoumi *et al*., 2015; WHO, 2022). Poor responders have a tendency for low antral follicle count, number of oocytes, fertilization rate, embryo cleavage rate, pregnancy rate and birth rate (Ubaldi *et al*., 2014; Ferraretti *et al*., 2011).

One of the etiologies of poor outcome in IVF in poor responder women is the unknown location of the meiotic spindle in the oocyte; thus, when ICSI is performed, it can affect the spindle itself. The meiotic spindle, by controlling chromosomal movements throughout the different stages of meiosis, plays a key role in the successful completion of meiosis. Disturbances in meiotic spindles have been suggested as predisposing oocytes to perturbation of chromosomal segregation and subsequent aneuploidy, maturation arrest, an increased incidence of cell death and subsequent lower fertilization rates (Kilani & Chapman, 2014)

Currently, poor responders are treated by stimulation to increase the number of oocytes retrieved (Ubaldi *et al*., 2014). Several studies have shown that the IVF-ICSI method may be used to increase the fertilization rate, cleavage rate, and pregnancy rate in this group (Namgoong & Kim, 2018; Farhi *et al.*, 2019). Hence, the optimization of the IVF-ICSI method is needed to produce embryos with good quality, despite the limited number of oocytes. Previous studies have shown that the meiotic spindle is not always aligned with the first polar body (PB) in metaphase II human oocytes. To date, Polarized Light Microscopy (PLM) has been used to find and locate the meiotic spindle to avoid disrupting it while injecting oocytes in IVF laboratories (Polyzos *et al*., 2014; Konc *et al*., 2004).

The observation of the meiotic spindle aims to prevent genetic material damage to the oocyte during the ICSI procedure. The disrupted meiotic spindle will reduce the accuracy of chromosomal segregation, genomic stability and meiotic division. Visualization of the meiotic spindle in oocytes can assist in predicting the quality of the oocytes and the potential for embryonic development (Swiatecka *et al*., 2014; Yang *et al.*, 2020).

The aim of this study is to evaluate the relationship between meiotic spindle viewing and IVF-ICSI outcomes in poor responder women.

## MATERIALS AND METHODS

### Study Design and Participants

This is a retrospective analytical study involving 115 poor responder patients who underwent IVF-ICSI cycles at the Halim Fertility IVF Center IVF from January-November 2019. All the patients were eligible if they fulfilled the inclusion criteria based on the Bologna criteria (Ferraretti *et al*., 2011). Exclusion criteria in this study were patients with pre-existing medical conditions, endometriosis cyst, Polycystic Ovary Syndrome (PCOS), fibroid, adenomyosis, severe male infertility with TMSC ≤5x10^6^, and patients with lost follow up.

**Figure 1:**
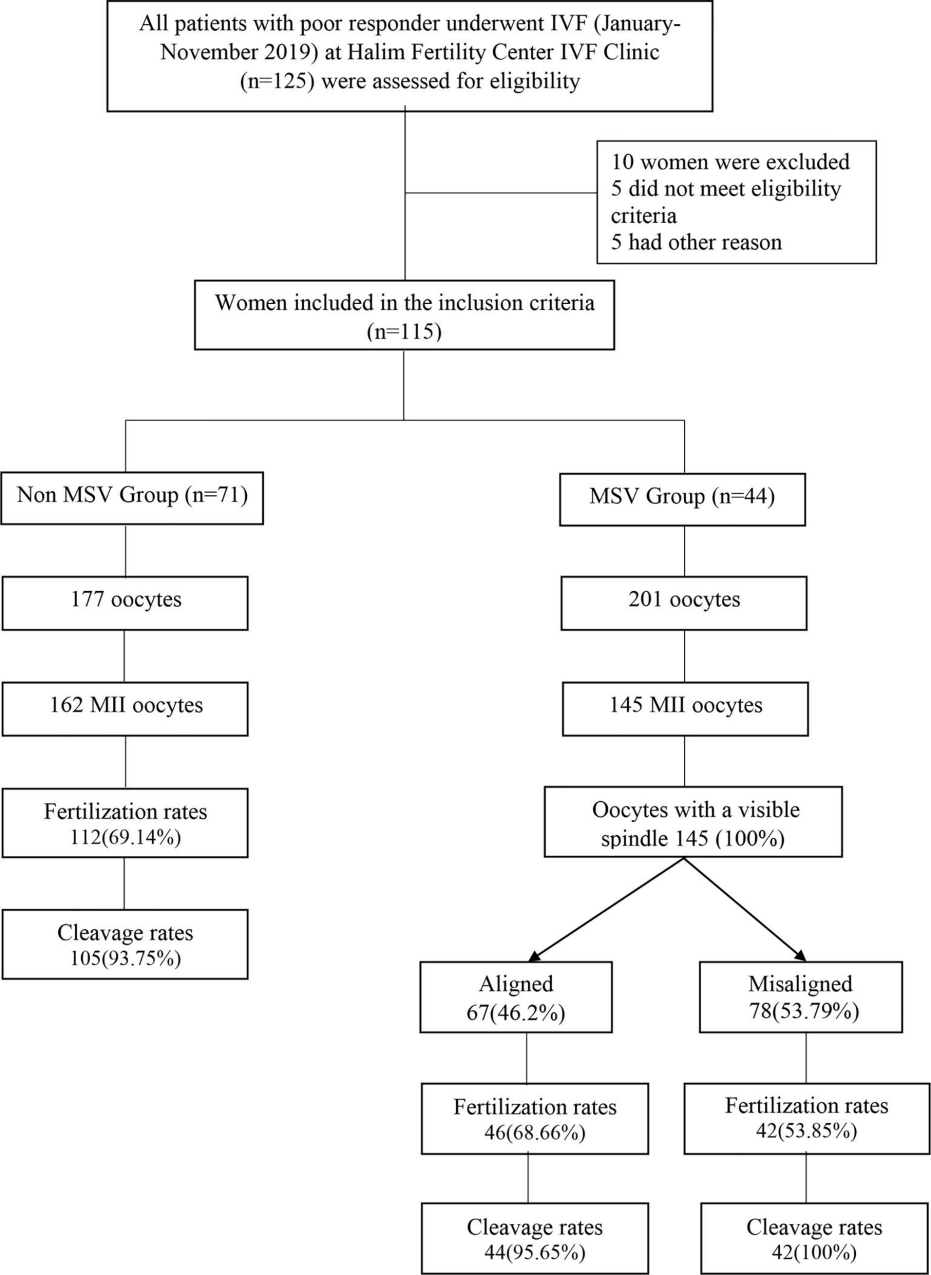
Schematic Study of Meiotic Spindle View and IVF outcome in poor responders.

The patients were divided into two groups: a control group without meiotic spindle viewing oocytes (non-MSV) and a case group with meiotic spindle viewing oocytes (MSV). This study had been approved by the ethical committee of Stella Maris Women’s and Children’s Hospital. All the patients agreed to share the outcomes of their own cycles for research purposes (Figure 1).

### Controlled Ovarian Stimulation

We stimulated our patients with the standard protocol for controlled ovarian stimulation, carried out using 150-300IU of recombinant FSH (Gonal-F, Merck KGaA, Germany) as daily dose, starting on day 2 or 3 of the cycle. We used a gonadotropin-releasing hormone (GnRH) antagonist (Cetrotide; Merck KGaA, Germany), starting when at least one follicle >14mm was visualized, at a dose of 0.25mg to prevent a premature luteinizing hormone (LH) surge. Follicular growth was monitored using transvaginal ultrasound, starting on day 6 of the gonadotropin administration daily. The average of stimulation duration was 10-12 days. Oocyte maturation was triggered with recombinant hCG (Ovidrel 250-500µg, Merck KGaA, Germany), when the lead follicles reached 17-18mm. Oocytes were retrieved 36-38 hours after the injection of hCG by transvaginal ultrasound guiding the needle aspiration of follicles.

### Oocyte Morphology Assessment

The aspirated follicular fluid was collected in a 14 ml polypropylene tube. The fluid was then poured into a 60mm Petri dish and examined under a light microscope to see cumulus oocyte complexes (COC). COC were separated and transferred to the petri dish containing a gamete buffer medium (Sydney IVF, Australia). The next step, the COC group was transferred to a 4-well petri dish to store and prepare for denudation.

The retrieved oocytes were maintained in a culture medium supplemented (G-IVFTM Plus, Vitrolife) with 10% protein and covered with paraffin oil for 2 to 3 hours before removing the cumulus cells. The surrounding cumulus cells were removed after exposure to an N-2-hydroxyethylpiperazine- N0-2-ethanesulfonic acid (HEPES)-buffered medium containing hyaluronidase (SynVitroTM Hyadase, Origio). The remaining cumulus cells were mechanically removed by gently pipetting with a hand-drawn Pasteur pipette. The COC were incubated at 37℃, 6% CO_2_ and 5% O_2_ for approximately 2 hours. Oocyte morphology was assessed just before sperm injection (4 hours after retrieval) using an inverted Nikon Diaphot (stereomicroscope Nikon smz 1000+) microscope.

### Sperm Preparation

Semen samples were obtained from the patient’s husband by masturbation after 3-5 days of ejaculatory abstinence. After liquefaction at room temperature, the samples were analyzed based on the World Health Organization (WHO) 2021 standards (WHO, 2021). The samples were then processed using a density gradient technique and were centrifuged for 5 minutes at a speed of 1400 rpm. The supernatant was removed and the pellet was mixed with 0.5 ml of wash medium (Sydney IVF, Australia). The pellets were incubated at 37℃ until they were used for ICSI.

### ICSI (Intracytoplasmic Sperm Injection) and injection position adjustment

The COC were denudated to get a clear view of the oocyte. The maturation stage of the cycles (metaphase II) was observed by the appearance of the first polar body (PB). The ICSI dish contained 8 drops of 15µl of culture medium (Origio, Denmark), 1 drop of 10µl of polyvinylpyrrolidone (Origio, Denmark) for sperm preparation and layered with culture oil. The ICSI dish was incubated at 37℃ until the ICSI procedure. The injection needles and holder pipettes were attached to the micromanipulator. Oocytes from MSV group were observed under polarized light microscopy (PLM) and oocytes from non-MSV group were observed under light microscope.

The ICSI procedure was performed by injecting one normal sperm into one mature oocyte. The oocytes from the non-MSV group were microinjected at 90^o^ angle with the first polar body (PB) and were positioned at the 6 or 12 o’clock position without observing the meiotic spindle position. The oocytes of the MSV group were observed with polarized light microscopes (PLM) to visualize the meiotic spindle before the injection. We used polarized light microscopes (PLM) (Oosight, Hamilton). Immediately before the ICSI procedure, the oocytes were screened by using polarized light microscopes (PLM), to observe and visualize the meiotic spindle and avoid disrupting it. The alignment method for spindle was differentiated if the first polar body was aligned with the meiotic spindle and misaligned if the first polar body was not aligned with the meiotic spindle. The first polar body and meiotic spindle were positioned at the 6 or 12 o’clock position and were microinjected at 90^o^ angle. The fertilized oocytes were transferred to a petri dish containing culture medium (Origio, Denmark) and were incubated at 37^o^C, 6% CO_2_ and 5% O_2_.

### Embryo Morphology Assessment

The fertilized oocytes were checked for embryo development using a light microscope. The fertilization rate was checked 17±1 hours after ICSI and indicated by the appearance of 2 pronuclei. Embryo quality assessment was carried out on the 3rd day (cleavage stage) (68±1 hours after ICSI). Scoring measurements based on the Istanbul Consensus were divided into 3 grade groups in the study. Embryo grade 1 (good) has an equal blastomere size and fragmentation of <10%, Grade 2 (fair) has an unequal blastomere size with the fragmentation of 10-25% and Grade 3 (poor) has an unequal blastomere size with the fragmentation >25%.

### Statistical analysis

We used the Kolmogorov-Smirnov test to assess normality of the sample quantitative variables. The data was nonparametric and baseline comparisons were made with the Mann-Whitney U test. The Mann Whitney test was used to analyze demographic characteristics data. The Mann Whitney was also used to analyze the MSV group parameters and outcomes of IVF-ICSI between the MSV and non-MSV groups. Analyses were performed using the Statistical Package for Social Sciences (SPSS) version 20.0 (Chicago, IL, USA). A *p*-value of <0.05 was considered statistically significant.

## RESULTS

From January until November 2019, we included 115 poor responder women. Table 1 presents the characteristics of participants. The average age of participants with MSV group was 37.73±1.90 years and there was no significant difference involving the age of women between the MSV and the non-MSV groups (*p*=0.076). The average duration of infertility in the MSV group was 9.76±4.31 years and there were significant differences in the duration of infertilities between the MSV and the non-MSV groups (*p*=0.023). The average body mass index (BMI) in the MSV group was 25.36±4.48 and there were significant differences in the duration of infertility between the MSV and the non-MSV groups (*p*=0.049). In this study, the average of Antral Follicle Count (AFC) in the MSV group was 4.68±1.02, and there was no significant difference in the AFC between the MSV and the non-MSV groups (*p*=0.796). The average of Anti Mullerian Hormone (AMH) in the MSV group was 0.85±0.21 and there was no significant difference in the AMH between MSV and non MSV groups (*p*=0.547).

**Table 1:** The participants’ demographic data.

Variables	Non MSV group (n=71)	MSV group (n=44)	-value
Female Age (years)	38.7±3.29	37.73±1.90	0.076
Infertility duration (years)	10.35±5.00	9.76±4.31	0.023
Body Mass Index	26.81±3.98	25.36±4.48	0.049
Underweight	18.36±0	18.34±0.02	0.480
Normal weight	22.86±1.51	22.35±1.65	0.385
Overweight Obese	26.66±1.27	27.07±1.18	0.245
	32.06±2.19	34.41±3.65	0.188
AFC (mean±SD)	4.73±1.03	4.68±1.02	0.796
AMH (ng/mL) (mean±SD)	0.82±0.21	0.85±0.21	0.547

* *Mann-Whitney U test*.

### Morphology and meiotic spindle

**Figure 2:**
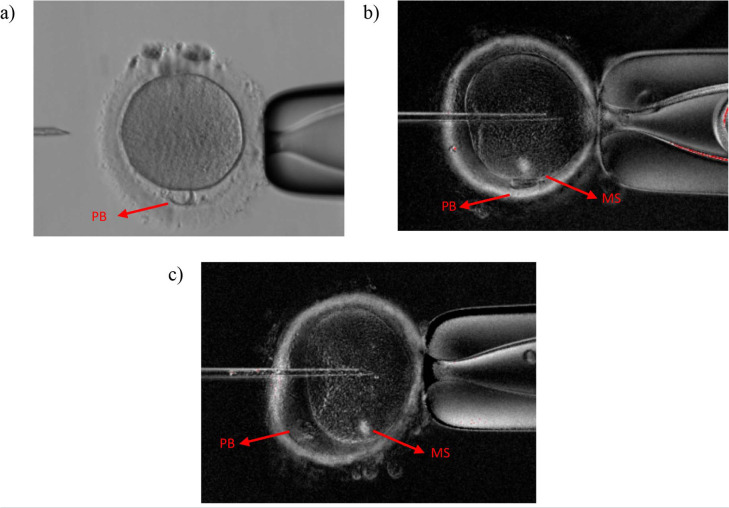
Oocytes were visualized under a microscope: a) light microscope, b) polarized light microscope with meiotic spindle above PB and c) polarized light microscope with misaligned meiotic spindle. PB= Polar body, MS= Meiotic Spindle.

We had 378 oocytes retrieved from OPU and they were randomly distributed to the non-MSV group (n=177) and the MSV group (n=201). After denudation, as many as 162 and 145 mature oocytes were found in the non-MSV group and in the MSV group, respectively (Table 2). All mature oocytes were sorted out and prepared to undergo ICSI. The meiotic spindle of the mature oocytes in the MSV group were detected by using polarized light microscopes (Figure 2).

**Table 2:** The MSV group parameters.

Variables	Aligned (n=67)	Misaligned (n=78)	*p*-value
Location of spindle (%)	46.2	53.79	
Fertilization rate	74.29±35.03	53.39±42.29	0.029^*^
Cleavage rate	95.16±19.81	100	0.229

* *Mann-Whitney U test.*

From Table 2, in the MSV groups, we found the location of spindle with aligned PB was 46.2% and misaligned PB was 53.79%. We found that the fertilization rate in aligned PB was higher than misaligned and there was significant difference between the two groups (*p*=0.029). The cleavage rate in misaligned PB was higher than among the aligned PB and there was no significant difference between the two groups (*p*=0.229).

In Table 3, we found that the cycle duration and total gonadotropin dose were comparable between non-MSV and MSV groups and there was no significant difference between the two groups (*p*=0.230; *p*=0.153). The number of oocytes retrieved was higher in the MSV group than the non-MSV group, and there was a significant difference between the two groups (*p*=0.015). The number of mature oocytes (MII) was higher in the MSV group than in the non-MSV group, and there was no significant difference between the two groups (*p*=0.111). The fertilization rate was higher in the non-MSV group, compared to the MSV group, and there was no significant difference between the two groups (*p*=0.596). The embryo cleavage rate was higher in the MSV group than in the non- MSV group and there was a significant difference between the two groups (*p*=0.019). The implantation rate was higher in the MSV group than in the non-MSV group, and there was no significant difference between the two groups (*p*=0.056). The clinical pregnancy rate was higher in the MSV group than in the non-MSV group, and there was a significant difference between the two groups (p=0.044). We also found that grade 1 and grade 2 Day-3 embryo count was higher in the non-MSV group than in the MSV-group (60% *vs*. 40%; 53% *vs*. 47%), but there was no significant difference between the two groups (*p*=0.708). We found that grade 3 Day-3 embryo count was higher in the non-MSV group than in the MSV group (35.24% *vs*. 31.40%), and there was no significant difference between the two groups (*p*=0.708).

**Table 3:** Outcome of IVF-ICSI between MSV and Non-MSV Group.

Variables	Non MSV group (n=71)	MSV group (n=44)	*p*-value
Cycle duration (days)	8.7±1.60	9.2±1.50	0.230
Total Gonadotropin Dose (IU)	2732±1.2	2692±1.10	0.153
Number of Oocyte Retrieved (mean±SD)	82.49±1.72	4.57±4.19	0.015
Number of Mature Oocyte (MII; mean±SD)	2.28±1.59	3.30±2.72	0.111
Fertilization Rate (mean±SD)	70.13±38.68	68.09±31.77	0.596
Embryo Cleavage Rate (mean±SD)	78.91±38.83	93.94±21.89	0.019
Implantation rate (n, %)	5/71 (7.04%)	4/44 (9.1%)	0.056
Clinical Pregnancy rate (n, %)	16/71 (22.5%)	11/44 (25%)	0.044
Day 3 Embryo Grading Grade 1 Grade 2 Grade 3	6 (60%) 62 (53%) 105 (55%)	4 (40%) 55 (47%) 27 (42.2%)	0.708

* *Mann-Whitney U test.*

From Table 4, we found that grade 1 Day-3 embryo count was higher in the non-MSV group than in the MSV group (5.71% *vs*. 4.65%). We found that grade 2 Day-3 embryo count was higher among the MSV group than in the non-MSV group (63.95% *vs*. 59.05%). We found that grade 3 Day-3 embryo count was higher in non-MSV group than in the MSV group (35.24% *vs*. 31.40%).

**Table 4:** Day-3 Embryo grading between Non-MSV and MSV groups.

	Grade 1	Grade 2	Grade 3
Non-MSV Group	6 (5.71%)	62 (59.05%)	37 (35.24%)
MSV Group	4 (4.65%)	55 (63.95%)	27 (31.40%)

## DISCUSSION

Poor responders had the criteria of having few oocytes (< 3 follicles), advanced age (> 38 years old), decreased follicular response, and low estradiol level (<500pg/ml) (Ferraretti *et al*., 2011). Based on the bologna criteria, at least two of the following three criteria must be present to classify a patient as a poor responder, (i) advanced maternal age, (ii) previous POR after ovarian stimulation, and (iii) abnormal ovarian reserve test (Ferraretti *et al*., 2011). Oocyte quality is the major cause of reduced implantation with advancing maternal age and the clearest link so far between maternal age and embryo competence is aneuploidy (ESHRE Special Interest Group of Embryology & Alpha Scientists in Reproductive Medicine, 2017). Oocyte quality has been regarded as a variable that may influence embryo quality (ESHRE Special Interest Group of Embryology & Alpha Scientists in Reproductive Medicine, 2017; Rienzi *et al*., 2003). Lubis *et al.* (2017) found that the highest incidence of aneuploidy embryo was observed in poor oocyte quality followed by moderate oocyte and good oocyte quality. These conditions gave limitations in oocyte selection, hence affecting the development and quality of embryos (Ubaldi *et al.*, 2014). The polarized light microscope is used to observe the meiotic spindle to improve the ICSI in poor responder patients including fertilization rate, cleavage rate, and clinical pregnancy rate (Namgoong & Kim, 2018; Farhi *et al*., 2019; Konc *et al*., 2004). Disturbances of meiotic spindles have been suggested as predisposing oocytes to perturbation of chromosomal segregation and subsequent aneuploidy, maturation arrest, an increased incidence of cell death, and subsequent lower fertilization rates. Halim *et al.* (2017) found that fertilization rates, cleavage rates, and high-quality embryos were not affected by abnormal oocyte morphology but the implantation rate was found to be higher in normal morphology oocytes than abnormal morphology oocytes.

In this study, the fertilization rate did not show significant differences between the two groups. Where the fertilization rate of the MSV group has a percentage of 70.13±38.68% and the non-MSV group has a percentage of 68.09±31.7%. The fertilization rate in this study did not have a significant difference and might be caused by various things such as high numbers of misalignment in the MSV group and meiotic spindle size. The position of the polar body had a major effect on the fertilization rate. It showed a significant difference (*p*=0.029) between the above PB group and the misaligned group with the values of 74.29±35.03 and 53.39±42.29, respectively. Rienzi *et al.* (2003) showed that the high distance between the spindle position and the polar body had an association with abnormal oocytes that cause failure in second meiotic anaphase. This will affect the fertilization rate decrease in the MSV group. The low fertilization rate can also be caused by the size of the oocyte spindle.

According to a previous study, Tomari *et al.* (2018) found that spindle size influences fertilization rate, blastocyst rate, and pregnancy rate. Spindle sizes of 90-120µm^2^ had a high fertilization rate, blastocyst rate, and pregnancy rate compared to larger or smaller sizes.

Normally, the location of the spindle is directly above the polar body. In the other case, the location of the polar body does not always predict the precise location of the spindle. The presence of meiotic spindles in ICSI procedures can also determine the optimal position for sperm injection in oocytes and give good outcomes from ICSI. The use of the meiotic spindle view aims to prevent spindle and chromosome damage at the time of ICSI (Konc *et al*., 2004; Swiatecka *et al*., 2014).

In this study, the meiotic spindle view showed a significant improvement in cleavage rate and embryo quality. The embryo cleavage showed significant differences between the non-MSV and the MSV groups (^p^=0.019). In the MSV group, the embryo cleavage rate was higher (93.94±21.89) compared to the non-MSV group (78.91±38.83). The difference found from the data showed the treatment could increase the cleavage rate more than the non-MSV group. The non-MSV group had a low cleavage rate because the ICSI procedure is only based on polar body position and there is a chance for spindle disruption at the time of the procedure (Ubaldi *et al*., 2014).

The meiotic spindle viewing can also prevent the occurrence of genetic material damage to the oocyte during the ICSI procedure (Konc *et al*., 2004). In this study, there was no significant difference in the cleavage rate between the aligned and misaligned groups (*p*=0.229). The meiotic spindle view will prevent poor outcomes from the development of embryos in the MSV group and be able to prevent embryo development disruption due to misaligned meiotic spindles (Petersen *et al.,* 2009; Konc *et al*., 2004; Swiatecka *et al*., 2014). The results from this study are also supported by the study by Asa *et al*. (2017) who showed the results of embryo cleavage rate in the embryo group with the meiotic spindle view method is higher than without meiotic spindle observation (Tomari *et al.*, 2018).

The meiotic spindle plays an important role in embryonic development. Spindles function in chromosomal segregation in the meiotic phase. When the chromosomes are impaired, the meiotic process will be disrupted and cause aneuploidy or genomic imbalances in embryos. Thus, the damage to the meiotic spindle will lead to poor outcomes in development and fertilization. Meiotic spindle visualization before ICSI is recommended to be performed in ART procedures, and it could be a marker of oocyte quality and embryo development (Petersen *et al.,* 2009; Swiatecka *et al*., 2014; Yang *et al.,* 2020).

**Figure 3:**
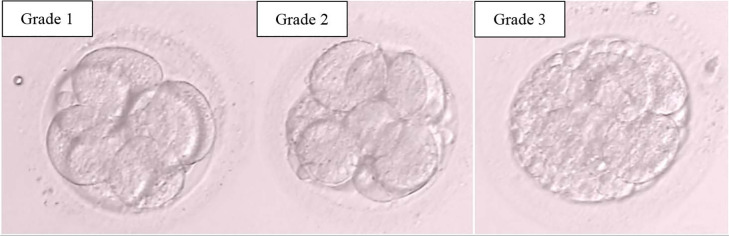
Day 3 Embryo grading: Grade 1 (left), Grade 2 (middle), Grade 3 (right).

**Figure 4:**
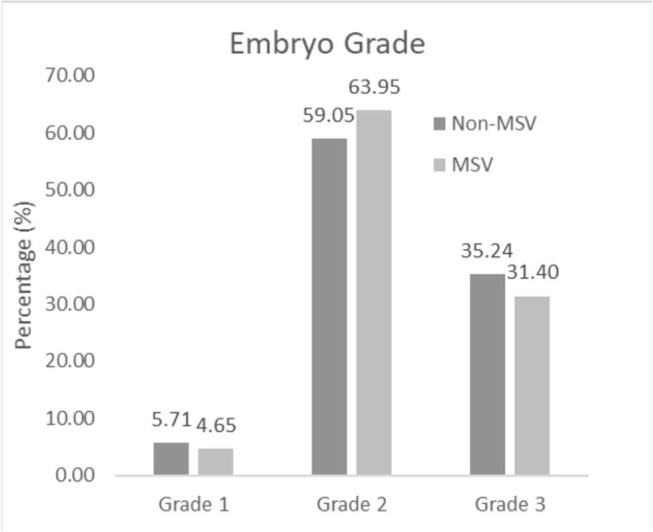
Percentage of Day 3 Embryo Grading in non-MSV and MSV groups.

The good quality of embryos was higher in the MSV group than in the non-MSV group. The majority of results of the study were grade 2 (59.05% and 63.95%), followed by grade 3 (35.24% and 31.40%) compared to grade 1 (5.71% and 4.65%) in both groups (Figure 3 and Figure 4). The percentage of grade 1 patients was low due to the patients being poor responders. Poor responders tend to have poor embryo quality related to the poor oocyte quality retrieved (Ubaldi *et al.,* 2014). Although grade 1 had a lower percentage, the MSV group improved the grade 2 percentage and decreased the grade 3 percentage. This result showed that the quality of embryos had improved outcomes by carrying out the meiotic spindle view. Madaschi *et al.* (2008) also found that meiotic spindle visualization in oocytes was able to predict the potential fertilization rate, embryo development, and better clinical outcomes. Asa *et al.* (2017) found that meiotic spindle results had a greater percentage in grade 2 compared to the group without meiotic spindle view.

The main limitation of this study is that it is a retrospective study, so that data sampling was not well controlled. Sampling is small in size and not well controlled. Clinical data, for instance live birth rate, was not analyzed because most of our patients in this study were lost to follow-up, because they lived outside of the city and the information regarding live birth rate could not be retrieved.

In conclusion, the meiotic spindle view might improve the outcome of IVF in poor responder women. Meiotic spindle observation has a positive association with embryo cleavage rate and quality of embryo in poor responder patients.
